# Developing Neurons Form Transient Nanotubes Facilitating Electrical Coupling and Calcium Signaling with Distant Astrocytes

**DOI:** 10.1371/journal.pone.0047429

**Published:** 2012-10-11

**Authors:** Xiang Wang, Nickolay Vassilev Bukoreshtliev, Hans-Hermann Gerdes

**Affiliations:** University of Bergen, Department of Biomedicine, Bergen, Norway; University of Waterloo, Canada

## Abstract

Despite the well-documented cooperation between neurons and astrocytes little is known as to how these interactions are initiated. We show here by differential interference contrast microscopy that immature hippocampal neurons generated short protrusions towards astrocytes resulting in tunneling nanotube (TNT) formation with an average lifetime of 15 minutes. Fluorescence microscopy revealed that all TNTs between the two cell types contained microtubules but 35% of them were F-actin negative. Immunolabeling against connexin 43 showed that this gap junction marker localized at the contact site of TNTs with astrocytes. Using optical membrane-potential measurements combined with mechanical stimulation, we observed that ∼35% of immature neurons were electrically coupled with distant astrocytes via TNTs up to 5 hours after co-culture but not after 24 hours. Connexin 43 was expressed by most neurons at 5 hours of co-culture but was not detected in neurons after 24 hours. We show that TNTs mediated the propagation of both depolarization and transient calcium signals from distant astrocytes to neurons. Our findings suggest that within a limited maturation period developing neurons establish electrical coupling and exchange of calcium signals with astrocytes via TNTs, which correlates with a high neuronal expression level of connexin 43. This novel cell-cell communication pathway between cells of the central nervous system provides new concepts in our understanding of neuronal migration and differentiation.

## Introduction

Over the past decade intensive work focusing on the communication between neurons and astrocytes has led to a change in the long-standing view that astrocytes provide only structural and trophic support for neuronal cells. Astrocytes for example were shown to be crucial for the maintenance of the microenvironment of mature neurons by clearing neurotransmitter from within the synaptic cleft [1]. Furthermore, it was demonstrated that astrocytes modulate the formation and function of synapses and neuronal excitability by releasing neuroactive compounds such as apoE/cholesterol particles, thrombospondins, glutamate, ATP and D-serine [2], [3]. In murine postnatal hippocampal slice cultures, direct physical contact between astrocytes and pyramidal neurons was found to play a role in synapse formation [4]. Particularly, the employment of fluorescent membrane markers in conjunction with time-lapse imaging demonstrated the highly dynamic nature of the astrocyte-neuron interaction involving ultrafine astrocytic processes and dendritic protrusions of neurons [4], [5], [6]. However, little is known about the interaction of immature neurons and astrocytes during the early stage of embryogenesis, especially before the establishment of membrane excitability and synapse formation.

Nano-scaled membrane connections were recently observed during developmental processes of various organisms. In mouse embryo, ectoderm cells localized to juxtaposed folds, were connected by long thin cellular bridges during neural tube closure [7]. Similarly, thin membrane connections were also found between epiblast cells during gastrulation in zebrafish embryo [8]. In fact, these membrane tubes were described as the underlying structure of a previously unrecognized cell-to-cell communication pathway and referred to as tunneling nanotubes (TNTs) [9] or membrane nanotubes [10]. To date, TNT-like structures have been documented in numerous cell lines such as fibroblasts, epithelial cells and immune cells [11], [12], as well as for primary cells including neurons [13] and astrocytes [14], [15]. Furthermore, it was shown that TNT-like structures facilitate the transfer of diverse cellular components ranging from cytoplasmic molecules such as calcium ions to vesicles of endosomal origin and mitochondria [11], [12]. Also pathogens such as the human immunodeficiency virus (HIV) [16], [17] and prions [13], [18] were found to spread between cells through these tubes. Recently, depolarization signals were shown to diffuse through TNTs resulting in opening of low voltage-gated calcium channels in the receiving cells [19], [20]. Importantly, the electrical coupling depended on the presence of gap junctions interposed at the membrane interface between TNT and the connected cell. These TNTs were characterized by the absence of dye coupling and a small conductance compared to the normal gap junction connections. Most likely the unique biophysical character of the TNT determined these properties [19], [20]. Considering the widespread occurrence of TNT-like structures and their multi-faceted functions in cell-to-cell communication, it is conceivable that they are also implicated in the crosstalk between astrocytes and neurons during brain development. To address this issue, we co-cultured immature neurons with astrocytes and probed for TNT-dependent electrical coupling. Our data show that TNTs indeed form between both cell types and facilitate electrical coupling and intercellular calcium signaling, which is accompanied by a high expression level of neuronal connexin 43 (Cx43).

## Results

### Immature Neurons Form Tunneling Nanotube-like Connections with Astrocytes

**Figure 1:**
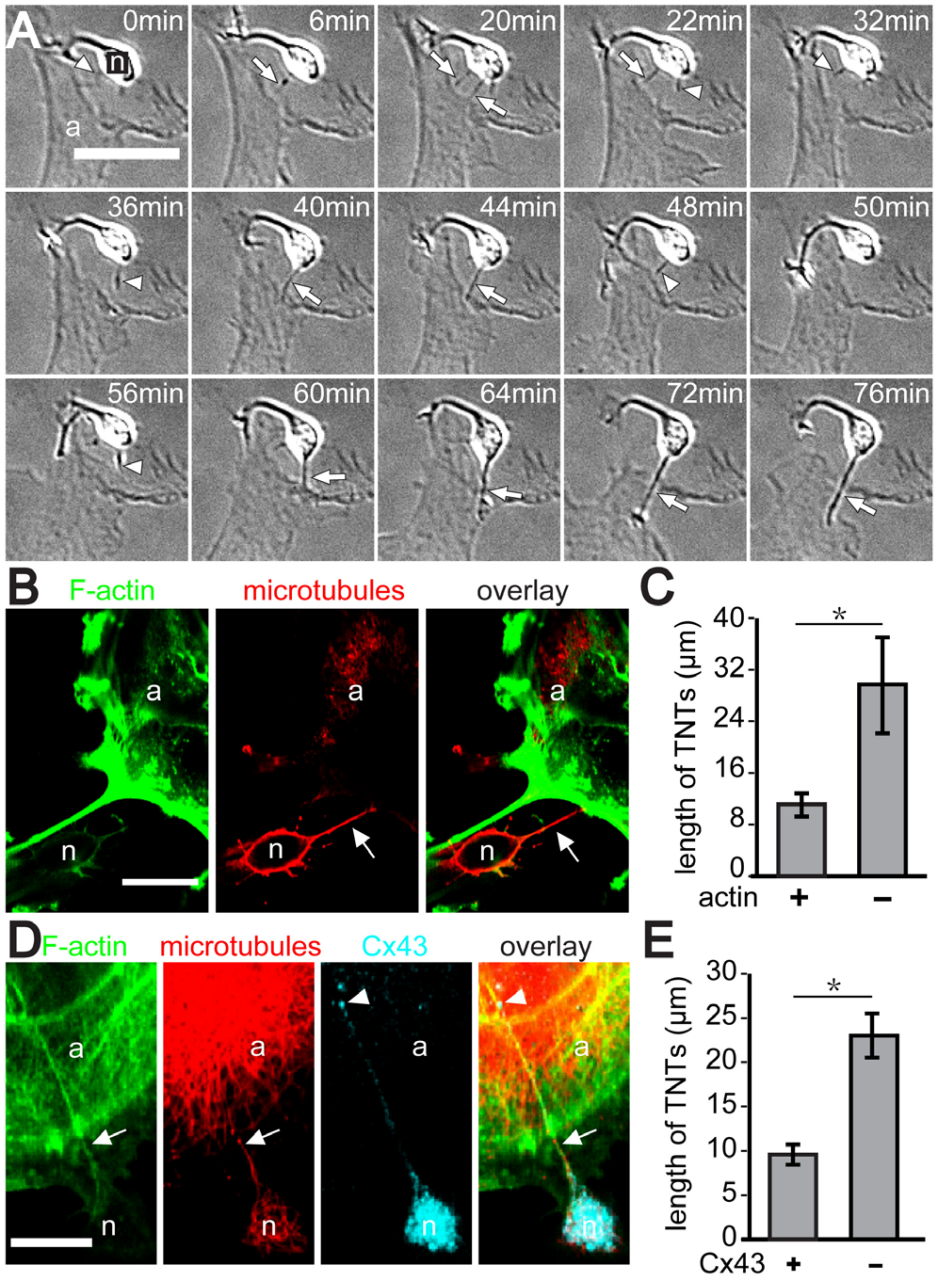
Formation of TNT-like structures between neurons and astrocytes. (**A**) Neurons form tubular extensions that connect to astrocytes. Astrocytes were co-cultured with freshly prepared dissociated hippocampal neurons and imaged by DIC microscopy 2 hours later. Shown are selected time frames depicting the formation of TNT-like structures (*arrows*) between a neuron (“n”) and an astrocyte (“a”). Note that the neuron displays tubular extensions before TNT formation and after their breakage (*arrowheads*). (**B**) A portion of TNT-like structures is microtubules positive but deficient in F-actin. The confocal fluorescence images depict a TNT (*arrow*) between neuron (“n”) and astrocyte (“a”), which contains microtubules (*middle*) but no F-actin (*left*). (**C**) TNTs positive for F-actin are shorter than those lacking F-actin. (**D**) TNT-like structures display Cx43 immunofluorescence signals. Neurons and astrocytes were co-cultured for 5 hours, fixed and fluorescently labeled using phalloidin-Alexa Fluor (*green*) and antibodies against α-tubulin (*red*) and Cx43 (*cyan*). The confocal image shows a typical TNT (*arrow*) formed between a neuron (“n”) and an astrocyte (“a”). Note the F-actin and microtubule staining of the TNT along with punctate Cx43 immunofluorescence signals at the astrocyte-neuron contact site (*arrowhead*). (**E**) TNTs positive for Cx43 are shorter than those lacking Cx43. The graphs represent a statistical analysis of 20 TNT structures (C and E). Data were analyzed by student's two-tailed t-test. Error bars show mean ± SEM. Scale bars = 20 µm.

To investigate if immature neurons can form TNTs with astrocytes, freshly prepared hippocampal neurons from fetal E18 Wistar rats were co-cultured with a low-density cell population of astrocytes. This ensured that a sufficient number of neurons grew in close proximity to individual astrocytes. TNT formation was monitored by differential interference contrast microscopy (DIC) 2 hours after start of the co-culture. This precluded the formation of neurites and their potential interference with the analyses. From the movies that were acquired we observed that occasionally non-polarized neurons, identified by their small diameter and phase brightness, generated short protrusions towards astrocytes resulting in TNT-like connections between the two cell types (movie S1 and selected frames in Figure 1A). In all observed cases (n = 12), TNTs were formed by neurons that grew in close proximity (less than 10 µm) to astrocytes. Furthermore, from 7 analyzed neurons with a distance of less than 10 µm to astrocytes, 6 grew protrusions specifically towards the astrocytes resulting in TNT formation (7 movies within a 2 hour time frame of co-culturing). These TNT structures formed transiently and displayed a lifetime of 14.8±7.9 min (mean ± SD, n = 12 TNTs, mean length = 7.1 µm). Their breakage always occurred at the end facing the astrocyte followed by retraction of the protruded end towards the neuron, and finally they disappeared. Furthermore, our data clearly showed that TNTs are formed in one direction only, because astrocytes were never observed to generate membrane protrusions towards developing neurons.

To address which cytoskeletal elements were present inside TNTs connecting neurons and astrocytes, the co-cultures were stained with fluorescently labeled phalloidin and a monoclonal antibody against α-tubulin to detect F-actin and microtubules, respectively. Subsequent fluorescent microcopy showed that all analyzed TNTs were positive for microtubules (n = 20, Figure 1B), which is different from the filopodial precursors of TNTs. However, 35% of TNTs did not show F-actin staining (Figure 1B). Interestingly, the TNTs lacking F-actin were longer (29.7±7.42 µm, mean ± s.e.m, n = 7) than TNTs containing F-actin (11.2±1.7 µm, mean ± s.e.m, n = 13) as shown in Figure 1C. Further analysis using a specific antibody against Cx43 revealed that a significant portion of TNTs (n = 7/20) displayed a punctate labeling of this marker, which was localized at the contact site of the TNT and the astrocyte (Figure 1D). In addition, all Cx43 positive TNTs (n = 7) contained F-actin and were shorter than TNTs without Cx43 (Figure 1E).

### Depolarization Signals Spread through TNT Connections between Immature Neurons and Distant Astrocytes

**Figure 2:**
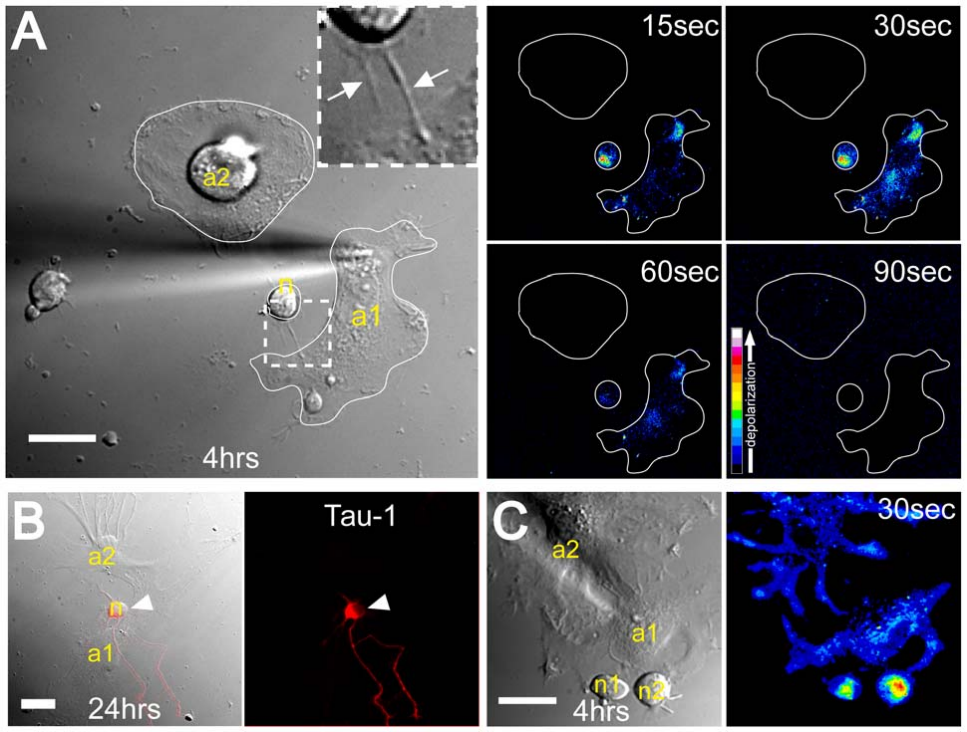
Depolarization signals spread between TNT-connected neurons and astrocytes. (**A**) Neurons and astrocytes are electrically coupled via TNTs. The DIC image shows the mechanically stimulated astrocyte (“a1”), the TNT-connected putative neuron (“n”), and a control cell (“a2”) after 4 hours of co-culture. The magnification box displays the TNTs (*arrows*). The pseudo-colored images, generated by subtraction of the image before stimulation from the respective images acquired after indicated times of stimulation, show the transient increase of DiBAC_4_(3) fluorescence after mechanical stimulation. The color bar indicates relative levels of depolarization. (**B**) After mechanical stimulation, the co-culture was continued for additional 24 hours. Then the cells were fixed and subjected to immunofluorescence analysis using an antibody against tau-1 (*red*). The DIC image shows the cells (“n”, “a1” and “a2”) at almost the same positions as in (A). The fluorescence image (*right*) depicts the tau-1 staining of neuron “n” in soma and dendrites (*arrowheads*). (**C**) Abutted cells display electrical coupling after 4 hours of co-culturing. The DIC image shows a close association of one astrocyte (“a2”) and two neurons (“n1” and “n2”). All three cells are electrically coupled as indicated by the increase of DiBAC_4_(3) fluorescence after 30 sec (pseudo-colored fluorescence image, *right*). Scale bars = 20 µm.

Previously we showed that the presence of Cx43 at one end of the TNT was essential for the transfer of electrical signals between distant cells [19]. To investigate if neurons and astrocytes are electrically coupled through TNTs, both cell types were co-cultured for 1 hour and then analyzed in the presence of the membrane potential sensitive dye bis-(1,3-dibutylbarbituric acid) trimethine oxonol (DiBAC_4_(3)). As a characteristic feature DiBAC_4_(3) increases its fluorescence signal with increasing membrane potential and its sensitivity to detect TNT-dependent electrical coupling was confirmed by patch-clamp measurements as described previously [19]. When an astrocyte (Figure 2A, “a1”) with a TNT-connection to a neuron (Figure 2A, “n”) was depolarized by mechanical stimulation, the DiBAC_4_(3) fluorescence of both astrocyte and neuron increased continuously over a period of ∼60 seconds (Figure 2A, pseudo-colored images). At the same time, a control cell (Figure 2A, “a2”) close to the stimulated cell but lacking physical connection did not display an increase in fluorescence excluding the possibility that depolarization was propagated via diffusion of molecular signals between cells. This indicated a TNT-dependent electrical coupling of the stimulated astrocyte and the neuron. To conclusively prove that the measured TNT-connected cell (“n”) was indeed a neuron, the analyzed cells were further co-cultured for a total of 24 hours and then probed with an antibody for the neuron-specific marker tau-1. The staining pattern obtained proved that this cell is of neuronal origin and had differentiated into a polarized neuron (Figure 2B). A subsequent analysis of 20 TNT-connected neuron/astrocyte cell pairs revealed that 7 pairs (35%) displayed electrical coupling. In comparison, the electrical coupling frequency of abutted cell pairs within 5 hours of co-culturing was also tested. The results showed that both astrocyte/astrocyte and astrocyte/neuron cell pairs frequently displayed a strong coupling (Figure 2C, “a1-a2” and “a1-n1/2″, respectively). Analysis of 18 abutted neuron/astrocyte pairs revealed a coupling frequency of 55%, which is higher than that of the TNT-based coupling. This higher coupling frequency of abutted versus TNT-connected cells is in agreement with data previously obtained from other cell types and may reflect a higher number of gap junction channels in the case of abutted cells [19].

**Figure 3:**
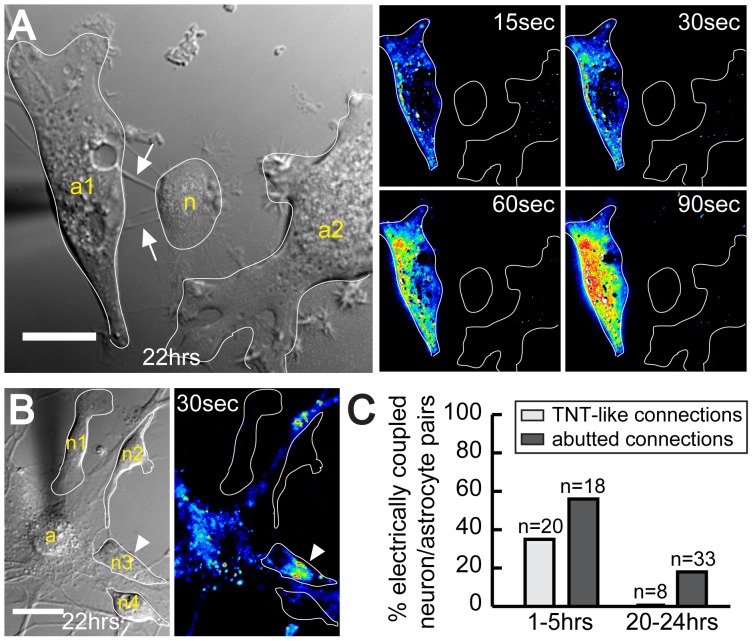
TNT-dependent electrical coupling between astrocytes and neurons declines during prolonged co-culturing. (**A**) A TNT-connected astrocyte-neuron cell pair did not show electrical coupling after 24 hours of co-culture. The DIC image depicts TNT-like structures (*arrows*) formed between neuron (“n”) and astrocyte (“a”) after 22 hours of co-culture. The neuron did not display depolarization when the astrocyte was stimulated (pseudo-colored fluorescence image, *right*). (**B**) Abutted neuron/astrocyte show less electrical coupling after 24 hours of co-culturing. The DIC image shows an astrocyte (“a”) closely associated with 4 neurons (“n1”, “n2”, “n3” and “n4”). Only one neuron (“n3”, *arrowhead*) was electrically coupled with the astrocyte as indicated by the increase of DiBAC_4_(3) fluorescence after 30 sec (pseudo-colored fluorescence image, *right*). (**C**) Electrical coupling ratio between neurons and astrocytes of abutted and TNT-connected cell pairs. Mechanical stimulation experiments were performed 1–5 or 20–24 hours after start of the co-culture. The percentage of electrically coupled neuron-astrocyte cell pairs (abutted or TNT-connected) was determined. “n”, number of cell pairs analyzed from at least three independent experiments. Scale bars = 20 µm.

To investigate whether or not the degree of electrical coupling between neurons and astrocytes changes with the progressive differentiation of neurons, we tested the electrical coupling after 24 hours of co-culture. At this time point hippocampal neurons have been classified as “stage 3” neurons [21]. Evidently, also at this stage TNT-like structures were detected between neurons and astrocytes (Figure 3A, arrows). However, all analyzed TNT-connected neuron/astrocyte pairs (n = 8) were not electrically coupled as illustrated in Figure 3A (pseudocolored images) and thus contrasted to our results obtained within 5 hours of co-culturing. To address if the absence of electrical coupling was only evident for TNT-specific connections, abutted neuron/astrocyte pairs were analyzed in parallel. Interestingly, also at these large contact sites, a decrease in electrical coupling was observed, down to 18% (n = 33, Figure 3B) after 24 hours as compared to 55% within 5 hours. Taken together, these data suggest that with progressive neuronal differentiation, the degree of electrical coupling decreases for both abutted and TNT-connected cell pairs (Figure 3C).

### Cx43 Expression Decreases during Neuronal Differentiation

**Figure 4:**
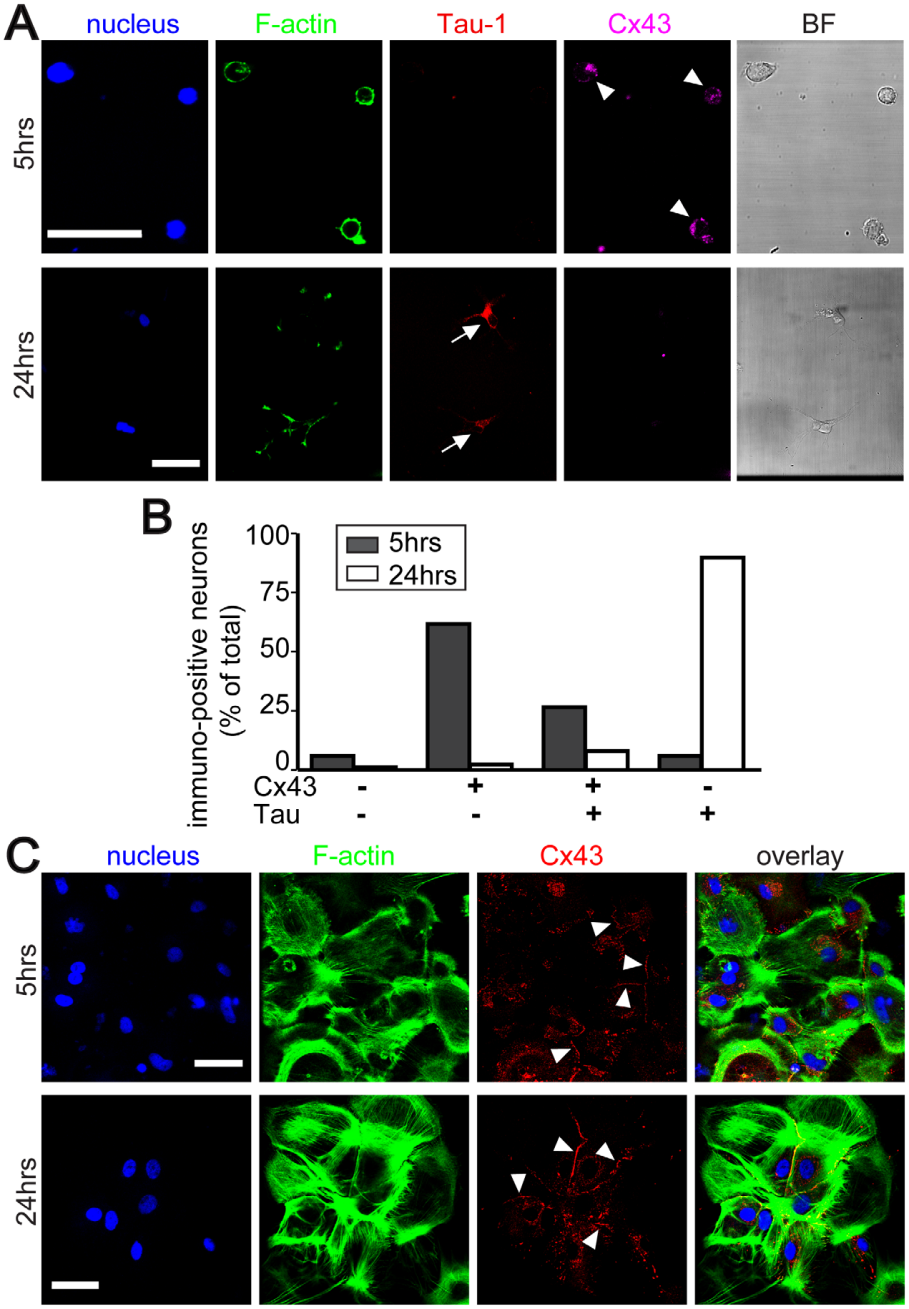
Expression of Cx43 in neurons declines with progressive maturation. (**A**) Cx43 is strongly expressed in immature neurons. Neurons were co-cultured with astrocytes for 5 or 24 hours, fixed and fluorescently stained with Hoechst 33342 (*blue*), phalloidin-Alexa Fluor (*green*), anti-tau-1 (*red*) and anti-Cx43 (*pink*). The right panels show corresponding bright field images. Single confocal sections show expression of Cx43 (*arrowheads*) in neurons after 5 hours of co-culture, but not after 24 hours. Note that at the same time more neurons became tau-1-positive (*arrows*) after 24 hours. (**B**) The expression level of Cx43 and tau-1 in neurons changes with their progressive maturation. Neurons after 5 hours (n = 68) or 24 hours (n = 89) of co-culturing were evaluated for Cx43 and/or tau-1-positive signals. The graph depicts the percentage of 4 different groups of neurons characterized by immunolabeling. (**C**) Cx43 is steadily expressed in astrocytes. Single confocal sections of cells labeled with Hoechst 33342 (*blue*), phalloidin-Alexa Fluor (*green*) and anti-Cx43 (*red*) show strong Cx43 immunoreactivity (*arrowheads*) in astrocytes after 5 and 24 hours of co-culture. Overlays are shown in right panels. Scale bars = 50 µm.

In agreement with our previous observation that TNT-mediated electrical coupling between NRK cells is Cx43-dependent [19], we speculated that the reduction in neuron-astrocyte coupling with prolonged culture time could be a decline in connexin expression. To test this hypothesis, we performed Cx43 immunofluorescence analyses after 5 and 24 hours of co-culturing. In parallel, neuronal differentiation was analyzed using the antibody against tau-1. Cx43 was strongly expressed by most neurons after 5 hours of co-culturing but was absent in nearly all cells after 24 hours (Figure 4A). In contrast, after 5 hours only few neurons were positive for tau-1, whereas after 24 hours the vast majority of them displayed a strong tau-1 staining in soma and dendrites (Figure 4A). A detailed analysis revealed that 88% of the neurons (n = 68) were Cx43 positive after 5 hours and of these only 32% of them were tau-1 positive (Figure 4B, black bars). After 24 hours of co-culture only 10% of the neurons (n = 47) remained positive for Cx43, whereas 98% displayed the neuron-specific tau-1 marker (Figure 4B, white bars). These results demonstrate a reversed trend in the expression profiles for both markers with increasing culture time, indicating a reduction in Cx43 expression during neuronal differentiation in the first 24 hours. For astrocytes, which were maintained for several weeks in culture, no significant change in the amount of surface-exposed Cx43 was observed during the first day (Figure 4C). Taken together, these data suggest that the decrease in electrical coupling between neurons and astrocytes is paralleled by the decline of the Cx43 level during neuronal differentiation.

### TNTs Mediate Depolarization and Calcium Signaling between Neurons and Astrocytes

**Figure 5:**
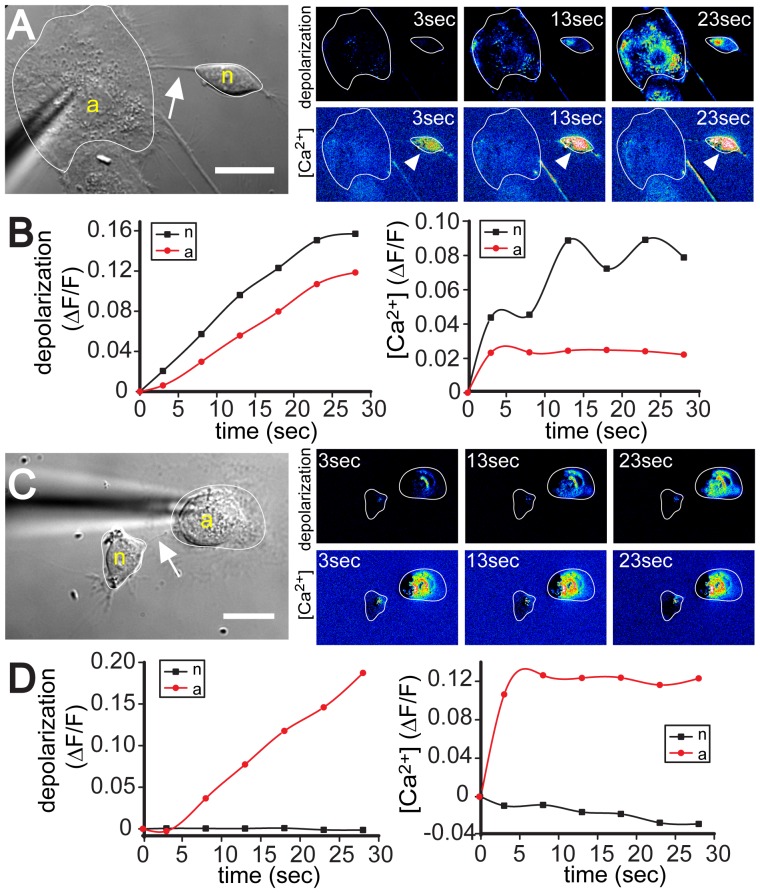
TNTs transmit depolarization and calcium signals to neurons. (**A, C**) The DIC images show the mechanically stimulated astrocytes (“a”), neurons (“n”), and the TNTs (*arrows*). The pseudo-colored intensity images, generated by subtraction of the image before stimulation, depict the fluorescence changes of DiBAC_4_(3) (*upper right panel*) and X-rhod-1 (*lower right panel*) at indicated times after mechanical stimulation. (**B**) Quantitative analysis of (A) demonstrates depolarization of the TNT-connected neuron (*left*, *black curve*) and the resultant increase in intracellular [Ca^2+^]_i_ (*right, black curve*). (**D**) Quantitative analysis of (C) shows that the TNT-connected neuron was neither depolarized (*left, black curve*) nor exhibited increased [Ca^2+^]_i_ (*right*, *black curve*). Scale bars = 20 µm.

Previously we reported that TNT-transmitted depolarization signals can activate low voltage-gated calcium channels resulting in transient calcium signals in the receiving cells [19]. To investigate whether depolarization of immature neurons is also accompanied by a rise in intracellular calcium ([Ca^2+^]_i_), we simultaneously measured changes in membrane potential with DiBAC4(3) and [Ca^2+^]_i_ with the fluorescent calcium indicator X-rhod-1 in 5-hour co-cultures. To exclude that the increase of [Ca^2+^]_i_ in TNT-connected neurons is the result of diffusible factors released from stimulated astrocytes, we used a cocktail of blockers (100 µM suramin to block purinergic receptors [22], 50 µM D-AP5 to block NMDA receptors [23] and 50 µM MCPG to block glutamate receptors [24]). Under these conditions, the mechanically stimulated astrocyte displayed depolarization and elevated [Ca^2+^]_i_ (Figure 5A). Importantly, the neuron connected to this astrocyte via a TNT also exhibited both depolarization and elevated [Ca^2+^]_i_ (n = 5, Figure 5A). A quantitative analysis revealed a simultaneous increase of the two signals in the neuron (Figure 5B). When astrocytes and neurons were not electrically coupled, no [Ca^2+^]_i_ increase was observed in the TNT-connected neuron even though [Ca^2+^]_i_ was strongly elevated in the astrocyte (n = 4, Figure 5C and D). Furthermore, we observed that membrane depolarization of a TNT-connected neuron did not always lead to changes in [Ca^2+^]_i_ (n = 2, Figure S1). Taken together, our results suggest that immature neurons can receive both electrical and calcium signals from distant astrocytes through TNT connections.

## Discussion

### Formation of TNT-like Structures

Live-cell imaging demonstrated that immature neurons initiate the formation TNT-like connections with astrocytes by a filopodia-based mechanism. This appears to be a directed process since all thin membrane protrusions emanating from neurons were directed towards astrocytes. It implies that chemical guidance cues through so far unknown substances secreted by astrocytes could play an important role in the guidance of membrane protrusions. The formation of TNT-like connections observed here is distinct from neurite formation. Pioneering work on developing hippocampal neurons showed that neurites establish substrate-associated membrane protrusions after 24 hours of culturing [25]. These initially short protrusions grow longer with increasing culturing time and develop into a prominent growth cone. In contrast, TNT formation is a substrate-independent process and can be observed already after 1 hour of culturing, resulting in transient cell-cell connections lasting for only 10–15 minutes.

Although the nanotubes bridging neurons and astrocytes display typical TNT morphology, their cytoskeletal composition differs significantly from TNTs formed by other cell types. First, all TNTs were positive for microtubules, hitherto only found in a subclass of TNTs of very few cell types such as macrophages and some NK cells [26], [27]. Second, although F-actin is considered as a hallmark of TNTs, not all TNTs connecting neurons and astrocytes did contain F-actin. This raises the question as to how the F-actin-deficient TNTs are formed. The fast elongation/retraction dynamics of thin membrane protrusions extending from neurons during the course of TNT formation suggests that F-actin may be involved in the initial event of TNT formation, but disassembles locally at a later stage, while microtubules remain inside nanotubes. Such a view concurs with our finding that F-actin was only found in short but not long TNTs. Alternatively, considering the finding of Yang *et al*. that actin filaments is not directly involved in protruding filopodia [28], microtubules may be critical for TNT formation between neurons and astrocytes. This interpretation is supported by our finding that all analyzed TNTs contained microtubules.

### Electrical Coupling via TNT-like Structures

Our data show that TNT-like structures bridging neurons and astrocytes frequently led to electrical coupling during the first few hours of co-culturing and disappeared after 24 hours, when neuronal development was already significantly advanced. In agreement with our results, dye transfer assays and electrophysiological measurements demonstrated that coupling of neurons from E18–E20 embryonic rats to the astrocytic monolayer was most frequent between day 2 and 3 and declined over the next 4 days [29]. This led to the concept that junctional communication between neuronal and astrocytic networks occurs predominantly during early embryonic development. The disappearance of heterocellular coupling with progressive cell differentiation is thought to be crucial for proper neuronal function since it prevents leakage of action potential from mature neurons to astrocytes. Subsequent work demonstrated that the underlying mechanism for the change in electrical coupling is the developmental regulation of connexin expression [30]. During the embryonic phase, a moderate expression of Cx43 was found in migratory neurons during neocortical brain development of E16–E19 rats and in conditionally immortalized mouse hippocampal multi-potent progenitor cells (MK31) [31], [32]. In addition, it was reported that Cx26 is also expressed in immature neurons [31], [33]. We therefore cannot exclude the possibility that Cx26 contributes to the documented electrical coupling. With progressive differentiation, hippocampal neurons were shown to shut down Cx43 expression and to form only gap junctions with themselves but no longer with astrocytes [34]. This is consistent with our data showing that expression of Cx43 is apparent for immature neurons and declines with progressive neuronal differentiation. Thus, TNTs failed to facilitate heterocellular electrical coupling between neurons and astrocytes, most likely due to the absence of interposed gap junctions at later developmental stages.

### Potential Implications of TNT-connectivity

We observed that TNTs were sufficient to transmit simultaneous depolarization and calcium signals to connected neurons. Importantly, we demonstrate that calcium signaling in neurons was absent when they were not electrically coupled with astrocytes despite the high level of [Ca^2+^]_i_ in the stimulated astrocytes. On one hand, these data do not support a simple model of calcium diffusion through TNTs but rather suggests a local influx of calcium ions into neurons through activated low-voltage calcium channels. In support of this, a special L-type calcium channel was found in hippocampal neurons of E18 embryos, which could be activated at low depolarization thresholds [35], [36]. On the other hand, the lack of a calcium signal in some depolarized neurons may be due to cell-to-cell variation of the expression levels of low-voltage calcium channels in our hippocampal cultures. Alternatively, the necessary threshold potential to activate the respective channels may not be always be reached through the TNT-mediated depolarization.

Calcium signals have been considered as important regulators of proliferation, migration and differentiation of neurons [37]. In particular, migration of interneurons is controlled by calcium influx through activation of voltage-sensitive calcium channels regulated by the potassium/chloride exchanger [38]. Since neuronal progenitor cell migration is accomplished by functional cooperation between neuronal and astrocytic networks [39], [40], it is conceivable that neurons may receive instructive cues for their migration via TNT-dependent signaling from astrocytes. Besides calcium signaling, it has been reported that Cx43-specific gap junctions of newborn neurons function as adhesion sites along radial glia fibres during the migration of neurons to the cortical plate [31]. Because radial glial cells share many characterizers with astrocytes [41], [42], immature neurons may also form connexin-positive TNTs to probe for the anchorage sites on radial glia cells and thereby increase the efficiency of the migratory process. Given that TNTs were shown to facilitate the intercellular exchange of molecules and organelles [20], morphogens or other factors relevant in development may also transfer via TNTs between astrocytes and neurons. These considerations suggest that the novel long-range transient interaction between neurons and astrocytes described here could shed new light on the mechanism of the documented roles of astrocytes in neurogenesis and neuronal migration during morphogenesis.

## Methods and Materials

### Cell Culture

Primary whole-brain astrocytes and hippocampal neurons from fetal E18 Wistar rats were prepared as described in accordance with the European Community Council Directive of 24 November 1986 (86/609/EEC) and approved by the Norwegian Committee for Animal Research [21], [43]. Astrocyte cultures were maintained in DMEM supplemented with 10% fetal bovine serum (Invitrogen, Carlsbad, CA, USA) for up to 4 weeks by passaging the cultures 1–2 times a week. For co-culture experiments, astrocytes (4.2×10^3^ cells/cm^2^) were first plated out in 35 mm glass bottom dishes (MatTek Corp., Ashland, MA, USA) coated with 0.1 mg/ml poly-l-lysine (Sigma-Aldrich Co., St. Louis, MO, USA) and 10 µg/ml laminin (Sigma-Aldrich). One hour after plating the astrocytes were stained with 10 µM CellTracker™ Blue CMAC (Invitrogen) for 30–45 min and washed with medium. Freshly prepared hippocampal neurons (3.3×10^3^ cells/cm^2^) were then plated directly to the stained astrocyte cultures in N-MEM supplemented with B27 neurobasal supplement (Invitrogen) and PSN antibiotic mix (Invitrogen) conditioned on astrocyte cultures for 3 days.

For time-lapse differential interference contrast (DIC) imaging, freshly prepared neurons were seeded on pre-plated astrocyte cultures. When the neurons were completely attached (1 hour after plating), the co-culture was imaged at 1 frame/2 min over a period of 2 hours using an Olympus IX70 microscope with a 40×/1.40 NA oil-immersion objective (Olympus Europa GmbH, Hamburg, Germany).

### Immunofluorescence

Co-cultures were fixed with 4% paraformaldehyde/4% sucrose in PBS 5 or 24 hours after plating. Immunofluorescence was performed according to standard procedures using the following primary antibodies: anti-tau-1 (Chemicon International, Temecula, CA, USA), anti-connexin-43 (Sigma-Aldrich) and anti-α-tubulin (Sigma-Aldrich). Actin was fluorescently labeled by Alexa Fluor 488™-coupled phalloidin (Invitrogen). Nuclei were stained by Hoechst 33342 (Invitrogen). Confocal imaging was performed on a Leica TCS SP5 confocal microscope (Leica Microsystems GmbH, Mannheim, Germany) equipped with a 40×/1.25 NA oil-immersion objective.

### Mechanical Stimulation and Membrane Potential Measurement

Mechanical stimulation experiments were performed 1–5 or 20–24 hours after the start of the co-culture. Co-cultures were pre-stained in DMEM supplemented with 2 µM of DiBAC_4_(3) (Sigma-Aldrich) for 30 min at 37°C and then replaced with pre-warmed fresh DMEM. Astrocytes and neurons were identified morphologically by DIC microscopy CellTracker Blue staining. Mechanical stimulations were applied to astrocytes in a chamber incubated at 37°C and 5% CO_2_, by microinjection using a FemtoJet/InjectMan NI 2 system (Eppendorf AG, Hamburg, Germany). Fluorescence images (16 bit) were acquired before and after mechanical stimulation with a 60×/1.40 NA oil-immersion objective, a Polychrome V monochromator (T.I.L.L. Photonics GmbH, Gräfelfing, Germany), and an Andor DU-885 camera controlled by IQ 7.0 software (Andor Technology, Belfast, Northern Ireland). CellTracker Blue and DiBAC_4_(3) were excited at 400 nm and 488 nm, respectively. Upon membrane depolarization cells display increased fluorescence of DiBAC_4_(3). The level of depolarization was expressed by pseudo-colored intensity images acquired with ImageJ software (http://rsbweb.nih.gov/ij/).

### Quantitative Imaging of Membrane Potential and [Ca^2+^]_i_

For simultaneous recordings of membrane potential and [Ca^2+^]_i_, 30 min co-cultures were loaded with 0.2 µM calcium indicator X-rhod-1 AM for 20 min, followed by a 30-min incubation in conditioned medium at 37°C supplemented with 4 µM DiBAC_4_(3), 100 µM suramin (Sigma-Aldrich), 50 µM D-AP5 (D(−)-2-Amino-5-phosphonopentanoic acid, Sigma-Aldrich) and 50 µM MCPG ((+)-α-Methyl-4-carboxyphenylglycine, Sigma-Aldrich). For two-wavelength imaging, the medium was replaced by colorless DMEM (Invitrogen) containing the three blockers mentioned above. The dye-loaded cells were excited at 488 nm and 560 nm and monitored under the same conditions as applied to single membrane potential measurements. Quantitative image analysis was performed with Image J. For quantitative analysis of DiBAC_4_(3) and X-rhod-1 images, the selected regions of interest (ROIs) and the mean fluorescence intensity of ROIs were calculated. The change of fluorescence intensity of cells (ΔF/F) was calculated as ΔF/F = (F_n_ − F_0_)/F_0_, where F_n_ is the mean fluorescence intensity at time frame n after mechanical stimulation, F_0_ is the original mean fluorescence intensity before mechanical stimulation.

## Supporting Information

Figure S1 **Electrically coupled neuron does not exhibit Ca^2+^ signal.** **(A)** The DIC image shows the mechanically stimulated astrocyte (“a”), neuron (“n”), and a TNT connecting them (*arrow*). The pseudo-colored intensity images, generated by subtraction of the image before stimulation, depict the fluorescence changes of DiBAC_4_(3) (*upper right panel*) and X-rhod-1 (*lower right panel*) at indicated times after mechanical stimulation. **(B)** The neuron was electrically coupled with the astrocyte (*left, black curve*) but did not show an increased [Ca^2+^]_i_ (*right, black curve*). Scale bar = 20 µm.
(TIF)

Movie S1 **Formation of TNT-like structures between neurons and astrocytes, related to** **Figure 1** **.** The establishment of TNT-like structures between neurons and astrocytes was monitored by DIC microscopy (see Methods). The movie shows the frequent and apparently directed formation of these transient structures (*arrows*) initiated by the neuron and connecting to the astrocyte (see also Figure 1A).
(AVI)

## References

[1] Bergles DE, Diamond JS, Jahr CE (1999) Clearance of glutamate inside the synapse and beyond. Curr Opin Neurobiol 9: 293–298. 10395570 10.1016/s0959-4388(99)80043-9

[2] Allaman I, Belanger M, Magistretti PJ (2011) Astrocyte-neuron metabolic relationships: for better and for worse. Trends Neurosci 34: 76–87. 21236501 10.1016/j.tins.2010.12.001

[3] Volterra A, Meldolesi J (2005) Astrocytes, from brain glue to communication elements: the revolution continues. Nat Rev Neurosci 6: 626–640. 16025096 10.1038/nrn1722

[4] Nishida H, Okabe S (2007) Direct astrocytic contacts regulate local maturation of dendritic spines. J Neurosci 27: 331–340. 17215394 10.1523/JNEUROSCI.4466-06.2007 PMC6672072

[5] Haber M, Zhou L, Murai KK (2006) Cooperative astrocyte and dendritic spine dynamics at hippocampal excitatory synapses. J Neurosci 26: 8881–8891. 16943543 10.1523/JNEUROSCI.1302-06.2006 PMC6675342

[6] Nestor MW, Mok LP, Tulapurkar ME, Thompson SM (2007) Plasticity of neuron-glial interactions mediated by astrocytic EphARs. J Neurosci 27: 12817–12828. 18032653 10.1523/JNEUROSCI.2442-07.2007 PMC6673300

[7] Pyrgaki C, Trainor P, Hadjantonakis AK, Niswander L (2010) Dynamic imaging of mammalian neural tube closure. Dev Biol 344: 941–947. 20558153 10.1016/j.ydbio.2010.06.010 PMC3873863

[8] Caneparo L, Pantazis P, Dempsey W, Fraser SE (2011) Intercellular bridges in vertebrate gastrulation. PLoS One 6: e20230. 21647454 10.1371/journal.pone.0020230 PMC3102083

[9] Rustom A, Saffrich R, Markovic I, Walther P, Gerdes HH (2004) Nanotubular highways for intercellular organelle transport. Science 303: 1007–1010. 14963329 10.1126/science.1093133

[10] Onfelt B, Nedvetzki S, Yanagi K, Davis DM (2004) Cutting edge: Membrane nanotubes connect immune cells. J Immunol 173: 1511–1513. 15265877 10.4049/jimmunol.173.3.1511

[11] Davis DM, Sowinski S (2008) Membrane nanotubes: dynamic long-distance connections between animal cells. Nat Rev Mol Cell Biol 9: 431–436. 18431401 10.1038/nrm2399

[12] Gerdes HH, Carvalho RN (2008) Intercellular transfer mediated by tunneling nanotubes. Curr Opin Cell Biol 20: 470–475. 18456488 10.1016/j.ceb.2008.03.005

[13] Gousset K, Schiff E, Langevin C, Marijanovic Z, Caputo A, et al (2009) Prions hijack tunnelling nanotubes for intercellular spread. Nat Cell Biol 11: 328–336. 19198598 10.1038/ncb1841

[14] Zhu D, Tan KS, Zhang X, Sun AY, Sun GY, et al (2005) Hydrogen peroxide alters membrane and cytoskeleton properties and increases intercellular connections in astrocytes. J Cell Sci 118: 3695–3703. 16046474 10.1242/jcs.02507

[15] Wang Y, Cui J, Sun X, Zhang Y (2011) Tunneling-nanotube development in astrocytes depends on p53 activation. Cell Death Differ 18: 732–742. 21113142 10.1038/cdd.2010.147 PMC3131904

[16] Sowinski S, Jolly C, Berninghausen O, Purbhoo MA, Chauveau A, et al (2008) Membrane nanotubes physically connect T cells over long distances presenting a novel route for HIV-1 transmission. Nat Cell Biol 10: 211–219. 18193035 10.1038/ncb1682

[17] Sherer NM, Lehmann MJ, Jimenez-Soto LF, Horensavitz C, Pypaert M, et al (2007) Retroviruses can establish filopodial bridges for efficient cell-to-cell transmission. Nat Cell Biol 9: 310–315. 17293854 10.1038/ncb1544 PMC2628976

[18] Langevin C, Gousset K, Costanzo M, Richard-Le Goff O, Zurzolo C (2010) Characterization of the role of dendritic cells in prion transfer to primary neurons. Biochem J 431: 189–198. 20670217 10.1042/BJ20100698

[19] Wang X, Veruki ML, Bukoreshtliev NV, Hartveit E, Gerdes HH (2010) Animal cells connected by nanotubes can be electrically coupled through interposed gap-junction channels. Proc Natl Acad Sci U S A 107: 17194–17199. 20855598 10.1073/pnas.1006785107 PMC2951457

[20] Wang X, Gerdes HH (2012) Long-distance electrical coupling via tunneling nanotubes. Biochim Biophys Acta 1818: 2082–2086. 21930113 10.1016/j.bbamem.2011.09.002

[21] Kaech S, Banker G (2006) Culturing hippocampal neurons. Nat Protoc 1: 2406–2415. 17406484 10.1038/nprot.2006.356

[22] Weissman TA, Riquelme PA, Ivic L, Flint AC, Kriegstein AR (2004) Calcium waves propagate through radial glial cells and modulate proliferation in the developing neocortex. Neuron 43: 647–661. 15339647 10.1016/j.neuron.2004.08.015

[23] Parpura V, Basarsky TA, Liu F, Jeftinija K, Jeftinija S, et al (1994) Glutamate-mediated astrocyte-neuron signalling. Nature 369: 744–747. 7911978 10.1038/369744a0

[24] Anwyl R (1999) Metabotropic glutamate receptors: electrophysiological properties and role in plasticity. Brain Res Rev 29: 83–120. 9974152 10.1016/s0165-0173(98)00050-2

[25] Dotti CG, Sullivan CA, Banker GA (1988) The establishment of polarity by hippocampal neurons in culture. J Neurosci 8: 1454–1468. 3282038 10.1523/JNEUROSCI.08-04-01454.1988 PMC6569279

[26] Onfelt B, Nedvetzki S, Benninger RK, Purbhoo MA, Sowinski S, et al (2006) Structurally distinct membrane nanotubes between human macrophages support long-distance vesicular traffic or surfing of bacteria. J Immunol 177: 8476–8483. 17142745 10.4049/jimmunol.177.12.8476

[27] Chauveau A, Aucher A, Eissmann P, Vivier E, Davis DM (2010) Membrane nanotubes facilitate long-distance interactions between natural killer cells and target cells. Proc Natl Acad Sci U S A 107: 5545–5550. 20212116 10.1073/pnas.0910074107 PMC2851811

[28] Yang C, Hoelzle M, Disanza A, Scita G, Svitkina T (2009) Coordination of membrane and actin cytoskeleton dynamics during filopodia protrusion. PLoS One 4: e5678. 19479071 10.1371/journal.pone.0005678 PMC2682576

[29] Froes MM, Correia AH, Garcia-Abreu J, Spray DC, Campos de Carvalho AC, et al (1999) Gap-junctional coupling between neurons and astrocytes in primary central nervous system cultures. Proc Natl Acad Sci U S A 96: 7541–7546. 10377451 10.1073/pnas.96.13.7541 PMC22122

[30] Nagy JI, Dudek FE, Rash JE (2004) Update on connexins and gap junctions in neurons and glia in the mammalian nervous system. Brain Res Rev 47: 191–215. 15572172 10.1016/j.brainresrev.2004.05.005

[31] Elias LA, Wang DD, Kriegstein AR (2007) Gap junction adhesion is necessary for radial migration in the neocortex. Nature 448: 901–907. 17713529 10.1038/nature06063

[32] Rozental R, Morales M, Mehler MF, Urban M, Kremer M, et al (1998) Changes in the properties of gap junctions during neuronal differentiation of hippocampal progenitor cells. J Neurosci 18: 1753–1762. 9465000 10.1523/JNEUROSCI.18-05-01753.1998 PMC6792627

[33] Nadarajah B, Jones AM, Evans WH, Parnavelas JG (1997) Differential expression of connexins during neocortical development and neuronal circuit formation. J Neurosci 17: 3096–3111. 9096144 10.1523/JNEUROSCI.17-09-03096.1997 PMC6573667

[34] Rash JE, Yasumura T, Dudek FE, Nagy JI (2001) Cell-specific expression of connexins and evidence of restricted gap junctional coupling between glial cells and between neurons. J Neurosci 21: 1983–2000. 11245683 10.1523/JNEUROSCI.21-06-01983.2001 PMC1804287

[35] Kavalali ET, Plummer MR (1996) Multiple voltage-dependent mechanisms potentiate calcium channel activity in hippocampal neurons. J Neurosci 16: 1072–1082. 8558236 10.1523/JNEUROSCI.16-03-01072.1996 PMC6578793

[36] Lipscombe D, Helton TD, Xu W (2004) L-type calcium channels: the low down. J Neurophysiol 92: 2633–2641. 15486420 10.1152/jn.00486.2004

[37] Spitzer NC (2006) Electrical activity in early neuronal development. Nature 444: 707–712. 17151658 10.1038/nature05300

[38] Bortone D, Polleux F (2009) KCC2 expression promotes the termination of cortical interneuron migration in a voltage-sensitive calcium-dependent manner. Neuron 62: 53–71. 19376067 10.1016/j.neuron.2009.01.034 PMC3314167

[39] Manent JB, Beguin S, Ganay T, Represa A (2011) Cell-autonomous and cell-to-cell signalling events in normal and altered neuronal migration. Eur J Neurosci 34: 1595–1608. 22103417 10.1111/j.1460-9568.2011.07867.x

[40] Valiente M, Marin O (2010) Neuronal migration mechanisms in development and disease. Curr Opin Neurobiol 20: 68–78. 20053546 10.1016/j.conb.2009.12.003

[41] Campbell K, Gotz M (2002) Radial glia: multi-purpose cells for vertebrate brain development. Trends Neurosci 25: 235–238. 11972958 10.1016/s0166-2236(02)02156-2

[42] Malatesta P, Hack MA, Hartfuss E, Kettenmann H, Klinkert W, et al (2003) Neuronal or glial progeny: regional differences in radial glia fate. Neuron 37: 751–764. 12628166 10.1016/s0896-6273(03)00116-8

[43] Banker G, Goslin K (1998) Culturing nerve cells. Cambridge, Mass.: MIT Press. xii, 666 p., 611 p. of plates p.

