# Supplementary figures and images for: Developing Neurons Form Transient Nanotubes Facilitating Electrical Coupling and Calcium Signaling with Distant Astrocytes
Source: PLoS One. 2012 Oct 11;7(10):e47429. doi: 10.1371/journal.pone.0047429 (PMC3469499; doi:10.1371/journal.pone.0047429)

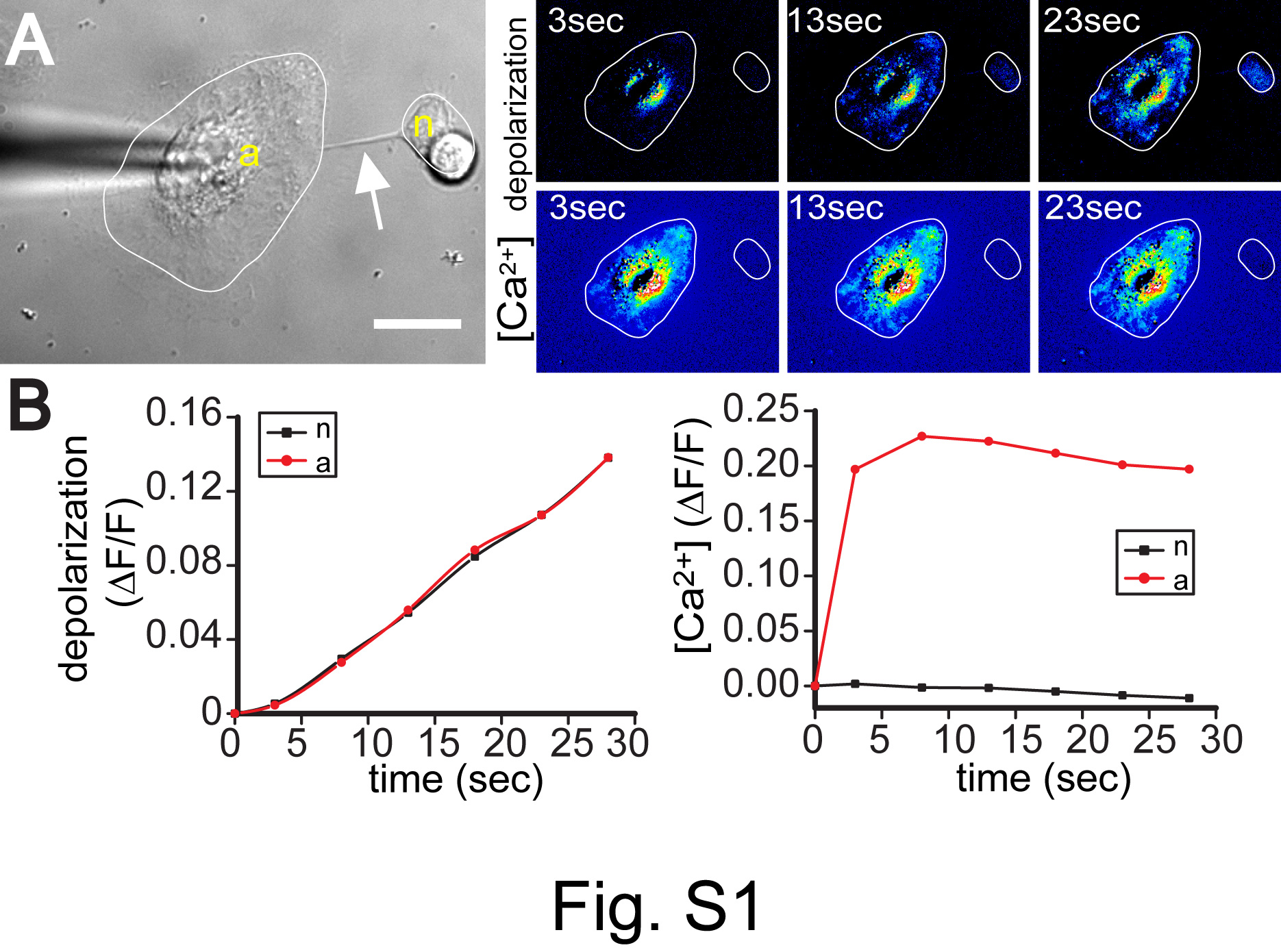

Supplement: Figure S1 — Electrically coupled neuron does not exhibit Ca2+ signal. (A) The DIC image shows the mechanically stimulated astrocyte (“a”), neuron (“n”), and a TNT connecting them (arrow). The pseudo-colored intensity images, generated by subtraction of the image before stimulation, depict the fluorescence changes of DiBAC4(3) (upper right panel) and X-rhod-1 (lower right panel) at indicated times after mechanical stimulation. (B) The neuron was electrically coupled with the astrocyte (left, black curve) but did not show an increased [Ca2+]i (right, black curve). Scale bar = 20 µm. (TIF) [file pone.0047429.s001.tif]
